# Evaluation of the WHO 2009 classification for diagnosis of acute dengue in a large cohort of adults and children in Sri Lanka during a dengue-1 epidemic

**DOI:** 10.1371/journal.pntd.0006258

**Published:** 2018-02-09

**Authors:** Champica K. Bodinayake, L. Gayani Tillekeratne, Ajith Nagahawatte, Vasantha Devasiri, Wasantha Kodikara Arachchi, John J. Strouse, October M. Sessions, Ruvini Kurukulasooriya, Anna Uehara, Shiqin Howe, Xin Mei Ong, Sharon Tan, Angelia Chow, Praveen Tummalapalli, Aruna D. De Silva, Truls Østbye, Christopher W. Woods, Duane J. Gubler, Megan E. Reller

**Affiliations:** 1 Department of Medicine, Faculty of Medicine, University of Ruhuna, Galle, Sri Lanka; 2 Division of Infectious Diseases, Department of Medicine, Duke University School of Medicine, Durham, NC, United States of America; 3 Duke Global Health Institute, Durham, NC, United States of America; 4 Department of Microbiology, Faculty of Medicine, University of Ruhuna, Galle, Sri Lanka; 5 Department of Pediatrics, Faculty of Medicine, University of Ruhuna, Galle, Sri Lanka; 6 Teaching Hospital Karapitiya, Galle, Sri Lanka; 7 Department of Pediatrics, Johns Hopkins University School of Medicine, Baltimore, MD, United States of America; 8 Emerging Infectious Diseases Program, Duke-NUS Medical School, Singapore; 9 Duke Ruhuna Collaborative Research Center, Faculty of Medicine, University of Ruhuna, Galle, Sri Lanka; 10 Genetech Research Institute, Colombo, Sri Lanka; 11 Department of Community and Family Medicine, Duke University School of Medicine, Durham, NC, United States of America; 12 Hubert-Yeargan Center for Global Health, Durham, NC, United States of America; 13 Division of Infectious Diseases, Department of Medicine, Johns Hopkins University School of Medicine, Baltimore, MD, United States of America; Institute of Collective Health, Federal University of Bahia, BRAZIL

## Abstract

**Background:**

Dengue is a leading cause of fever and mimics other acute febrile illnesses (AFI). In 2009, the World Health Organization (WHO) revised criteria for clinical diagnosis of dengue.

**Methodology/Principal findings:**

The new WHO 2009 classification of dengue divides suspected cases into three categories: dengue without warning signs, dengue with warning signs and severe dengue. We evaluated the WHO 2009 classification vs physicians’ subjective clinical diagnosis (gestalt clinical impression) in a large cohort of patients presenting to a tertiary care center in southern Sri Lanka hospitalized with acute febrile illness. We confirmed acute dengue in 388 patients (305 adults ≥ 18 years and 83 children), including 103 primary and 245 secondary cases, of 976 patients prospectively enrolled with AFI. At presentation, both adults and children with acute dengue were more likely than those with other AFI to have leukopenia and thrombocytopenia. Additionally, adults were more likely than those with other AFI to have joint pain, higher temperatures, and absence of crackles on examination whereas children with dengue were more likely than others to have sore throat, fatigue, oliguria, and elevated hematocrit and transaminases. Similarly, presence of joint pain, thrombocytopenia, and absence of cough were independently associated with secondary vs primary dengue in adults whereas no variables were different in children. The 2009 WHO dengue classification was more sensitive than physicians’ clinical diagnosis for identification of acute dengue (71.5% vs 67.1%), but was less specific. However, despite the absence of on-site diagnostic confirmation of dengue, clinical diagnosis was more sensitive on discharge (75.2%). The 2009 WHO criteria classified almost 75% as having warning signs, even though only 9 (2.3%) patients had evidence of plasma leakage and 16 (4.1%) had evidence of bleeding

**Conclusions/Significance:**

In a large cohort with AFI, we identified features predictive of dengue vs other AFI and secondary vs primary dengue in adults versus children. The 2009 WHO dengue classification criteria had high sensitivity but low specificity compared to physicians’ gestaldt diagnosis. Large cohort studies will be needed to validate the diagnostic yield of clinical impression and specific features for dengue relative to the 2009 WHO classification criteria.

## Author summary

Dengue is an important cause of acute fever in the tropics that is difficult to distinguish from other common etiologies of fever. The World Health Organization (WHO) revised criteria for the clinical diagnosis and classification of acute dengue in 2009. The performance of these criteria has not been widely evaluated in countries where dengue is endemic. We confirmed acute dengue in 388 of 976 patients presenting with acute febrile illness (AFI) to the largest tertiary care center in the Southern Province of Sri Lanka. We found specific clinical features and laboratory investigations to be predictive of acute dengue versus other AFI. The new WHO 2009 classification was more sensitive than physicians’ clinical diagnosis for identification of acute dengue on admission to hospital, but also over-estimated the severity of illness. Further large cohort studies are warranted to validate the performance of the 2009 WHO criteria for diagnosis and prognosis of dengue in regions where the disease burden is high.

## Introduction

Dengue is an important cause of morbidity and mortality worldwide in the tropics and mimics other causes of acute febrile illness (AFI) [1–3]. Early recognition of severe dengue could improve outcomes [4]. The 1997 World Health Organization (WHO) dengue clinical classification criteria were developed to assist with surveillance, triage, and treatment decisions in the management of dengue, but were difficult to apply clinically and had poor sensitivity in identifying severe dengue [5]. Therefore, World Health Organization (WHO) in 2009 revised the clinical criteria to improve the diagnosis of dengue [5]. The new criteria categorized patients with suspected dengue as having dengue without warning signs, dengue with warning signs, and severe dengue, and have improved sensitivity in identifying patients with severe dengue [6]. However, the performance of these revised criteria has not been widely evaluated in Sri Lanka [5]. To compare clinical features of dengue and to assess the performance of the revised 2009 WHO clinical criteria for the diagnosis of acute dengue, we prospectively studied a large cohort with AFI during an epidemic of dengue 1 (DENV-1) in southern Sri Lanka.

## Methods

### Febrile cohort

Trained study physicians prospectively enrolled patients with AFI admitted to the adult and pediatric wards of the largest (1,500 bed) tertiary care hospital in the Southern Province. Consecutive patients ≥1 year of age with documented fever (>38°C) at presentation or within 48 hours of hospital admission were eligible. We obtained epidemiologic and clinical data and an acute blood sample at enrollment and convalescent serum at 2–4 week follow-up. We recorded the treating physicians’ subjective clinical diagnosis at presentation. Study staff prospectively extracted all clinical data and the treating physicians’ clinical diagnosis at discharge from the patients’ medical records during hospitalization.

### Confirmation of acute dengue

Specimens were promptly frozen at -70°C and shipped on dry ice months later for off-site testing. We retrospectively confirmed acute dengue using IgG and IgM ELISA, virus isolation, RT-PCR for DENV, and RT-PCR for flaviviruses, as previously described [7]. Confirmed dengue was defined by 1) IgG seroconversion alone with positive IgM or IgM seroconversion (definitive serologic evidence), 2) PCR and either isolation or alternative target PCR (definitive virologic evidence), or 3) PCR and/or isolation with a positive convalescent IgM (virologic and serologic evidence). Primary and secondary acute dengue were determined by the absence or presence of IgG in the acute sample, respectively.

### Statistical analyses and definitions

The Chi square test or Fisher exact test were used for categorical variables and t-test or Kruskall-Wallis test for continuous variables. Bivariable logistic regression yielded odds ratios (OR) with 95% confidence intervals (95% CI) for features associated with acute dengue vs other AFI and secondary vs primary dengue. Multivariable logistic regression was performed for adults and children separately. All statistically significant (p<0.05) non-collinear clinical variables (symptoms and signs) were initially included (full model). For laboratory values, leukopenia (<4 x 10^3^ cells/μL, thrombocytopenia (<100 x 10^3^/μL), and elevated transaminases (AST or ALT > 120 IU, 3 times upper limit of normal) were included in the models rather than absolute laboratory values. For hematocrit and hemoglobin, absolute values were used given variability in standard values between males and females and adults and children. Variables were then sequentially removed to yield the most parsimonious model (p-value for all <0.05). Excluded variables were then reintroduced and retained if significant.

Both application of the WHO classification criteria and diagnostic testing for dengue were performed retrospectively for the purpose of the study. The treating physicians’ clinical diagnosis both at presentation and discharge reflected his or her subjective clinical impression. Laboratory confirmation of acute dengue was used as the gold standard for evaluating the accuracy of the 2009 WHO classification criteria and physicians’ clinical diagnoses at admission and discharge. To evaluate the performance of the treating physicians’ clinical diagnosis versus the WHO classification criteria at identifying acute dengue, sensitivity, specificity, positive predictive value, and negative predictive value were calculated. Receiver-operating characteristic (ROC) curves were generated and area under the curve (AUC) was calculated. STATA, version 11 (STATACorp, College Station, Texas) was used for all analyses.

### Ethical review

Ethical approval was obtained from Ethics committee of Faculty of Medicine University of Ruhuna, Institutional review boards of Duke University USA, Duke-National University Singapore and Johns Hopkins University USA.

Written informed consent was obtained from all patients ≥ 18 years of age, parental informed consent was obtained from patients 1–17 years of age and additionally written assent was obtained from all those aged 12–17 years to participate in the study. Study doctors (MBBS) with pediatric experience obtained informed consent in a pre-designed consent form.

## Results

### Febrile cohort

We obtained convalescent sera from 877 (89.6%) of 976 patients enrolled; 628 (64.3%) were male and 306 (31.4%) <18 years. Acute dengue was confirmed in 388/976 (40%); 39 with inconclusive results were excluded. Of confirmed dengue, 103 (26.6%) cases were primary, 245 (63.1%) secondary, and 40 (10.3%) could not be classified because of insufficient acute sera. Among 351 virologically confirmed (dengue PCR or virus isolation positive) cases, 320 (91.2%) were DENV-1, 25 (7.1%) DENV-4, and 6 (1.7%) DENV-2, with similar proportions in adults vs. children [in children, 60 (92.3%) DENV-1, 2 (3.1%) DENV-4, and 2 (3.1%) DENV-2].

### Clinical features of dengue vs. other AFI

In both adults and children, patients with acute dengue were older than those with other AFI and more likely to have joint pain, muscle pain, anorexia, right upper quadrant tenderness, rash, leukopenia (<4 x 10^3^ cells/μL), thrombocytopenia (<100 x 10^3^/μL), and elevated (>3 times normal) transaminases (Table 1). Those with acute dengue were less likely than others to have cough and lung crackles. Additionally, adults with dengue were more likely to have vomiting than those with other AFI and children with dengue were more likely to report headache, rhinitis, sore throat, and abdominal pain.

**Table 1:** Comparison of clinical and laboratory features in febrile children and adults with and without acute dengue, southern Sri Lanka, 2012–2013.

	Adults			Children		
Clinical Characteristics	Acute dengue N = 305	No dengue N = 340	p-value	Acute dengue N = 83	No dengue N = 209	p-value
Age, yrs	34(25–45)	37(28–52)	**< .001**	11 (7–16)	5 (3–9)	**< .001**
Male	199 (65.3%)	227 (66.8%)	0.68	51 (61.5%)	129 (61.7%)	0.97
**Symptom**						
Days of fever	4 (3–6)	5 (3–7)	0.10	5 (3–6)	4 (3–6)	0.41
Headache	251 (82.6%)	271 (79.9%)	0.40	59 (74.7%)	101 (50.3%)	**< .001**
Rhinitis/congestion	30 (9.8%)	51 (15.0%)	0.05	19 (22.9%)	104 (50.0%)	**< .001**
Sore throat	51 (16.7%)	72 (21.2%)	0.15	20 (24.7%)	27 (13.4%)	**0.02**
Cough	108 (35.9%)	164 (49.6%)	**< .001**	31 (37.4%)	135 (64.9%)	**< .001**
Joint pain	226 (74.6%)	198 (58.8%)	**< .001**	41 (51.9%)	48 (24.4%)	**< .001**
Muscle pain	216 (71.1%)	212 (62.9%)	**0.03**	43 (54.4%)	52 (26.5%)	**< .001**
Anorexia	281 (92.4%)	294 (86.5%)	**0.01**	72 (86.8%)	152 (72.7%)	**0.01**
Abdominal pain	74 (24.3%)	69 (20.3%)	0.22	35 (43.2%)	49 (23.7%)	**0.001**
Vomiting	153 (52.2%)	133 (39.6%)	**0.002**	50 (61.7%)	104 (50.2%)	0.08
Diarrhea	40 (13.2%)	48 (14.2%)	0.72	3 (3.7%)	29 (13.9%)	**0.01**
Dysuria	31 (10.2%)	38 (11.2%)	0.38	9 (10.8%)	7 (3.4%)	**0.02**
Oliguria	39 (12.8%)	43 (12.7%)	0.52	12 (14.5%)	13 (6.2%)	**0.02**
Fatigue	236 (78.2%)	252 (74.8%)	0.32	71 (88.8%)	123 (60.6%)	**< .001**
**Signs**						
Temperature (C)	100 (99–101)	99 (98–101)	**< .001**	100 (99–101)	99 (98–100)	**< .001**
Heart rate / min	80 (72–92)	82 (76–96)	0.06	100 (84–104)	100 (88–110)	0.06
Systolic BP	110 (110–120)	110 (110–120)	**0.03**	100 (90–110)	100 (90–110)	0.09
Diastolic BP	70 (70–80)	70 (70–80)	0.07	70 (60–70)	60 (60–70)	0.06
Conjunctival injection	52 (17.1%)	68 (20.1%)	0.36	7 (8.4%)	8 (3.8%)	0.14
Pharyngeal erythema/exudate	27 (8.9%)	21 (6.2%)	0.23	5 (6.0%)	12 (5.7%)	1.00
Lymnphadenopathy	29 (9.8%)	38 (11.3%)	0.61	18 (21.7%)	43 (20.7%)	0.87
Jaundice	3 (1.0%)	10 (3.0%)	0.10	0	0	—
Lung crackles	10 (3.3%)	48 (14.1%)	**<0.001**	3 (3.6%)	38 (18.2%)	**0.001**
Right upper abdominal tenderness	45 (14.9%)	23 (6.8%)	**0.001**	14 (17.3%)	7 (3.4%)	**<0.001**
Hepatomegaly	23 (7.6%)	24 (7.1%)	0.88	6 (7.3%)	15 (7.2%)	1.00
Rash	56 (18.4%)	39 (11.5%)	**0.02**	20 (24.1%)	19 (9.1%)	**0.002**
Flushing	36 (11.8%)	11 (3.2%)	**< .001**	15 (18.1%)	5 (2.4%)	**< .001**
**Laboratory parameter**	**Median IQR**					
WBC per μL*	3.4 (2.5–5.3)	7.0 (4.7–10.6)	**< .001**	3.4 (2.3–7.2)	7.7 (6.1–11.2)	**< .001**
Leukopenia*	189 (62.4%)	55 (16.5%)	**< .001**	42 (54.6%)	15 (9.0%)	**< .001**
ANC^#^ per μL	2.2 (1.4–3.9)	5.0 (2.8–8.1)	**< .001**	1.7 (1.1–4.8)	4.9 (3.6–7.1)	**< .001**
ALC^^^ per μL	0.7 (0.5–1.0)	1.2 (0.8–1.7)	**< .001**	1.0 (0.6–1.8)	2.0 (1.3–3.1)	**< .001**
Hemoglobin (g/dl)	13.5 (12.4–14.6)	13.5 (12.1–14.7)	0.56	12.9 (12.2–13.5)	12.0 (11.1–12.9)	**0.002**
Hematocrit	40.3 (37.0–43.7)	40.1 (36.4–43.3)	0.57	38.7 (36.6–40.6)	36.0 (34.0–39.0)	**< .001**
Platelets x1000/ μL)*	109 (64–157)	174 (120–236)	**< .001**	120 (72–218)	238 (181–298)	**< .001**
Thrombocytopenia*	136 (44.9%)	56 (16.8%)	**< .001**	30 (39.0%)	7 (4.2%)	**< .001**
Elevated transaminases^+^	54 (17.7%)	34 (10.0%)	**0.004**	12 (14.5%)	1 (0.5%)	**< .001**
Antibiotics at enrollment	126 (41.3%)	216 (63.5%)	**< .001**	96 (45.9%)	28 (33.7%)	0.06

Median (range) for continuous variables or number (percentage) for categorical variables are listed in the table.

* Within 48 hrs of admission.

^#^Absolute neutrophil count.

^^^Absolute lymphocyte count.

^+^ within 7 days. Leukopenia: <4 x 10^3^ cells/μL. Thrombocytopenia: <100 x 10^3^/μL. Transaminases: AST or ALT > 120 IU, 3 times upper limit of normal).

On multivariable analyses, both adults and children with acute dengue were more likely than those with other AFI to have leukopenia and thrombocytopenia (Table 2). Additionally, adults with acute dengue were more likely than those with other AFI to have joint pain, higher temperatures, and absence of crackles on examination. Children with acute dengue were more likely than others to have sore throat, fatigue, oliguria, an elevated hematocrit (≥20% from baseline) and elevated transaminases.

**Table 2:** Multivariable analysis of features associated with dengue, performed in children and adults separately, southern Sri Lanka, 2012–2013.

	Adults		Children	
Characteristic	Unadjusted OR (95% CI)	Adjusted final model OR (95% CI)	Unadjusted OR (95% CI)	Adjusted final model OR (95% CI)
Age, yrs	0.98 (0.97–0.99)	0.99 (0.97–1.00)	—	—
Sore throat	—	—	2.13 (1.11–4.06)	4.43 (1.04–18.87)
Fatigue	—	—	5.13 (2.43–10.85)	10.80 (1.42–82.27)
Joint pain	2.06 (1.47–2.89)	1.72 (1.16–2.55)	—	—
Decreased urine output			2.57 (1.12–5.90)	5.32 (1.08–26.33)
Temperature	1.25 (1.11–1.39)	1.22 (1.07–1.39)	—	—
Lung crackles	0.21 (0.10–0.42)	0.36 (0.16–0.80)	—	—
Leukopenia	8.38 (5.78–12.14)	5.82 (3.89–8.73)	12.16 (6.07–24.36)	8.06 (2.64–24.56)
Thrombocytopenia	4.03 (2.79–5.81)	1.93 (1.25–2.97)	14.41 (5.95–34.90)	13.12 (2.86–60.17)
Elevated hematocrit	—	—	1.12 (1.03–1.22)	1.18 (1.03–1.36)
Elevated transaminases			35.15 (4.49–275.19)	43.99 (3.08–627.84)

Leukopenia: <4 x 10^3^ cells/μL, Thrombocytopenia: <100 x 10^3^/μL. Elevated transaminases: AST or ALT > 120 IU, 3 times upper limit of normal).

Patients with acute dengue were hospitalized longer than those with non-dengue AFI (median 5 vs. 4 days, p<0.001). However, disease severity overall was low. Nine adults showed signs of plasma leakage and 16 patients (14 adults, 2 children) signs of hemorrhage. Among the 9 adults with plasma leakage 2 were classified as severe dengue and 6 were classified as dengue with warning signs per the WHO 2009 criteria. Among 16 patients with signs of hemorrhage, 1 was classified as severe dengue and 11 were classified as dengue with warning signs. Two adults with acute dengue (2 with severe dengue) and 4 adults with other AFI (0.6% of total cohort) required care in an intensive care unit; no patients with acute dengue died.

### Primary versus secondary dengue

Acute dengue was predominantly associated with secondary dengue, and those with secondary dengue were older. On bivariable analysis, both adults and children with secondary dengue were more likely to have muscle pain and thrombocytopenia and to report a longer duration of fever than those with primary dengue (Table 3). Adults with secondary dengue were also more likely to have joint pain and flushing. Children with secondary dengue were more likely to have fatigue and leukopenia and less likely to have rhinitis/congestion. On multivariable analysis, presence of joint pain (3.02 [CI 1.62, 5.64]), thrombocytopenia (OR 2.05 [1.12, 3.75]), and absence of cough (OR 0.49 [0.28, 0.89]) were independently associated with secondary vs primary dengue in adults whereas no variables were different in children.

**Table 3:** Comparison of clinical and laboratory features of primary versus secondary dengue in children and adults, June 2012- May 2013, southern Sri Lanka.

	Adults			Children		
Characteristic	Primary dengue n = 69	Secondary dengue n = 203	p-value	Primary dengue n = 34	Secondary dengue n = 42	p-value
Age	30.3 (24.2–43.8)	34.1 (26.5–46.1)	0.11	10.2 (5.0–15.9)	13.3 (8.2–16.1)	0.19
Male	45 (65.2%)	135 (66.5%)	0.85	24 (70.6%)	22 (52.4%)	0.11
**Symptom**						
Days of fever	4 (3–5)	5 (3–6)	**0.01**	4 (2–5)	5 (5–6)	**< .001**
Rhinitis/congestion	10 (14.5%)	15 (7.4%)	0.08	12 (35.3%)	6 (14.3%)	**0.03**
Sore throat	11 (15.9%)	33 (16.3%)	0.95	11 (33.3%)	8 (19.1%)	0.16
Cough	33 (47.8%)	65 (32.5%)	**0.02**	14 (41.2%)	15 (35.7%)	0.63
Joint pain	41 (59.4%)	163 (81.1%)	**< .001**	13 (40.6%)	26 (61.9%)	0.07
Muscle pain	40 (58.5%)	157 (77.3%)	**0.003**	14 (43.8%)	28 (66.7%)	**0.049**
Anorexia	64 (92.8%)	186 (92.1%)	0.86	29 (85.3%)	38 (90.5%)	0.49
Abdominal pain	20 (29.0%)	46 (22.8%)	0.30	14 (41.2%)	18 (43.9%)	0.81
Vomiting	29 (42.7%)	110 (56.7%)	**0.046**	18 (52.9%)	28 (70.0%)	0.13
Diarrhea	7 (10.1%)	29 (14.4%)	0.37	3 (8.8%)	0 (0%)	0.05
Dysuria	5 (7.3%)	25 (12.3%)	0.25	5 (14.7%)	4 (9.8%)	0.51
Oliguria	10 (14.7%)	25 (12.3%)	0.61	2 (5.9%)	9 (22.0%)	0.05
Headache	60 (87.0%)	166 (82.2%)	0.36	21 (65.6%)	32 (78.1%)	0.24
Fatigue	56 (82.4%)	158 (78.6%)	0.51	26 (78.8%)	40 (97.6%)	**0.01**
**Signs**						
Temperature	100.6 (99.2–101.0)	99.8 (98.8–100.8)	**0.04**	99.5 (98.4–100.8)	100.3 (99.0–100.8)	0.27
Heart rate/ min	80 (76–88)	80 (72–92)	0.76	98 (88–110)	96 (82–100)	0.26
Systolic BP	110 (110–120)	110 (100–120)	0.15	100 (90–110)	110 (100–110)	0.04
Diastolic BP	70 (70–80)	70 (70–80)	0.44	60 (60–70)	70 (60–70)	0.24
Conjunctival injection	16 (23.2%)	30 (14.8%)	0.11	6 (17.7%)	0 (0%)	**0.005**
Pharyngeal erythema/ exudate	4 (11.8%)	1 (2.4%)	0.10	7 (10.1%)	17 (8.4%)	0.65
Lymphadenopathy	9 (13.4%)	18 (9.2%)	0.32	9 (26.5%)	7 (16.7%)	0.30
Jaundice	0 (0%)	2 (1.0%)	0.41	0	0	—
Lung crackles	6 (8.7%)	4 (2.0%)	0.01	2 (5.9%)	1 (2.4%)	0.44
Right upper abdominal tenderness	8 (11.8%)	32 (15.9%)	0.41	8 (24.2%)	6 (14.6%)	0.29
Hepatomegaly	6 (8.8%)	15 (7.5%)	0.73	3 (8.8%)	1 (2.4%)	0.22
Rash	6 (8.7%)	41 (20.2%)	0.03	6 (17.7%)	12 (28.6%)	0.27
Flushing	3 (4.4%)	28 (13.8%)	**0.03**	4 (11.8%)	10 (23.8%)	0.18
**Laboratory parameter**	**Median IQR**					
WBC per μL	3.5 (2.5–6.7)	3.4 (2.4–5.1)	0.29	7.3 (4.0–10.8)	2.5 (2.2–3.8)	**< .001**
Leukopenia*	39 (56.5%)	127 (62.9%)	0.35	7 (23.3%)	32 (78.1%)	**< .001**
ANC^#^ per μL	2.5 (1.5–4.7)	2.1 (1.3–3.7)	0.09	4.8 (1.7–6.2)	1.3 (0.9–2.0)	**< .001**
ALC^^^ per μL	0.7 (0.5–1.1)	0.7 (0.5–0.9)	0.67	1.2 (0.7–2.8)	0.8 (0.6–1.3)	**0.009**
Hemoglobin (g/dl)	13.7 (12.5–14.7)	13.6 (12.4–14.7)	0.63	12.9 (11.8–13.9)	12.9 (12.2–13.5)	0.91
Hematocrit	41.0 (37.0–44.1)	40.3 (37.1–43.7)	0.65	38.8 (35.5–40.6)	38.6 (37.2–41.0)	0.71
Platelets (x1000/ /μL)*	130 (88–175)	103 (60–150)	**0.003**	204 (139–261)	84 (59–124)	**< .001**
Thrombocytopenia*	21 (30.4%)	99 (49.0%)	**0.007**	6 (20.0%)	23 (56.1%)	**0.002**
Elevated transaminases^+^	2 (2.9%)	17 (8.4%)	0.12	1 (2.9%)	3 (7.1%)	0.42
Antibiotics at enrollment	34 (49.3%)	79 (38.9%)	0.13	11 (32.4%)	15 (35.7%)	0.76

* Within 48 hrs of Admission

^#^Absolute neutrophil count.

^^^Absolute lymphocyte count.

^+^ within 7 days. Leukopenia: <4 x 10^3^ cells/μL. Thrombocytopenia: <100 x 10^3^/μL. Elevated transaminases: AST or ALT > 120 IU, 3 times upper limit of normal). Number (proportions) listed in table except WBC and platelet count, which are listed as median (IQR).

Both patients with primary and secondary dengue were hospitalized for a median of 5 days (p = 0.53). All 9 adults with plasma leakage (2 were classified as severe dengue and 6 were classified as dengue with warning signs per WHO 2009 WHO criteria) had secondary dengue. Among 16 patients with hemorrhage (one classified as severe dengue and 11clasiified as dengue with warning signs), 3 had primary dengue (2 adults, 1 child) and 12 had secondary dengue (11 adults, 1 child). Two adults with dengue who required intensive care (both classified as severe dengue) had secondary dengue. No deaths were recorded.

Clinical diagnosis by attending physician vs 2009 WHO classification for identifying acute dengue

In the febrile cohort, the most common clinical diagnoses at admission were unspecified viral fevers (40.6%), dengue (28.8%), leptospirosis (12.3%), lower respiratory tract infections (9.1%), and upper respiratory tract infections (4.3%) and the most common clinical diagnoses at discharge were unspecified viral fevers (30.5%), dengue (27.6%), lower respiratory tract infections (9.6%), leptospirosis (9.6%), and upper respiratory tract infections (4.7%). Among confirmed dengue cases, dengue was the most common clinical diagnosis both at admission (49%) and at discharge (58%), followed by unspecified viral fever (37% at admission and 24% at discharge). The sensitivity and specificity of clinical diagnosis of dengue by the attending physician at discharge in adults vs children was similar and was higher for secondary vs primary dengue (Table 4). In adults, the sensitivity of clinical diagnosis at discharge for secondary dengue was 64% (95% CI 57–70) vs 49% (95% CI 39–59) for primary dengue. In children, the sensitivity of clinical diagnosis was 71.4% (95% CI 56.7–83.4) vs 24.4% (95% CI 12.4–40.3) for secondary vs primary infections, respectively. The overall accuracy of physicians’ clinical diagnosis on hospital admission was 67.1% (95% CI 64.2–70.0) and 75.2% (95% CI 72.5–77.9) at discharge. Dengue was erroneously diagnosed clinically in 15% of cases at admission and 7% at discharge.

**Table 4:** Performance characteristics of clinical diagnosis at discharge versus the 2009 WHO classification for diagnosis of acute dengue, southern Sri Lanka, 2012–2013.

		Sensitivity (95%CI)	Specificity (95%CI)	PPV (95%CI)	NPV (95%CI)
All patients	Clinician diagnosis at admission	49.0 (43.9–54.1)	85.3 (82.0–88.1)	70.1 (64.3–75.5)	70.3 (66.6–73.7)
	Clinician diagnosis at discharge	57.7 (52.6–62.7)	92.7 (90.2–94.7)	84.9 (79.9–89.0)	75.6 (72.2–78.8)
	WHO	76.0 (71.5–80.2)	64.9 (60.7–68.8)	60.5 (56.0–64.8)	79.3 (75.2–82.9)
Children	Clinician diagnosis at admission	50.6 (39.4–61.8)	90.4 (85.6–94.1)	67.7 (54.7–79.1)	82.2 (76.6–86.9)
	Clinician diagnosis at discharge	50.6 (39.4–61.8)	97.1 (93.9–98.9)	87.5 (74.8–95.3)	83.2 (77.9–87.7)
	WHO	69.9 (58.8–79.5)	75.1 (68.7–80.8)	52.7 (43.0–62.3)	86.3 (80.4–90.9)
Adults	Clinician diagnosis at admission	48.5 (42.8–54.3)	82.1 (77.6–86.0)	70.8 (64.1–76.9)	64.0 (59.3–68.5)
	Clinician diagnosis at discharge	59.7 (53.9–65.2)	90.0 (86.3–93.0)	84.3 (78.7–88.9)	71.3 (66.8–75.6)
				
	WHO	77.7 (72.6–82.3)	58.5 (53.1–63.8)	62.7 (57.6–67.6)	74.5 (68.9–79.7)
Primary dengue	Clinician diagnosis at admission	33.0 (24.1–43.0)	—	—	—
	Clinician diagnosis at discharge	37.9 (28.5–48.0)	—	—	—
	WHO	59.2 (49.1–68.8)	—	—	—
Secondary dengue	Clinician diagnosis at admission	55.1 (48.6–61.4)	—	—	—
	Clinician diagnosis at discharge	66.9 (60.7–72.8)	—	—	—
	WHO	83.7 (78.4–88.1)	—	—	—

**Fig 1:**
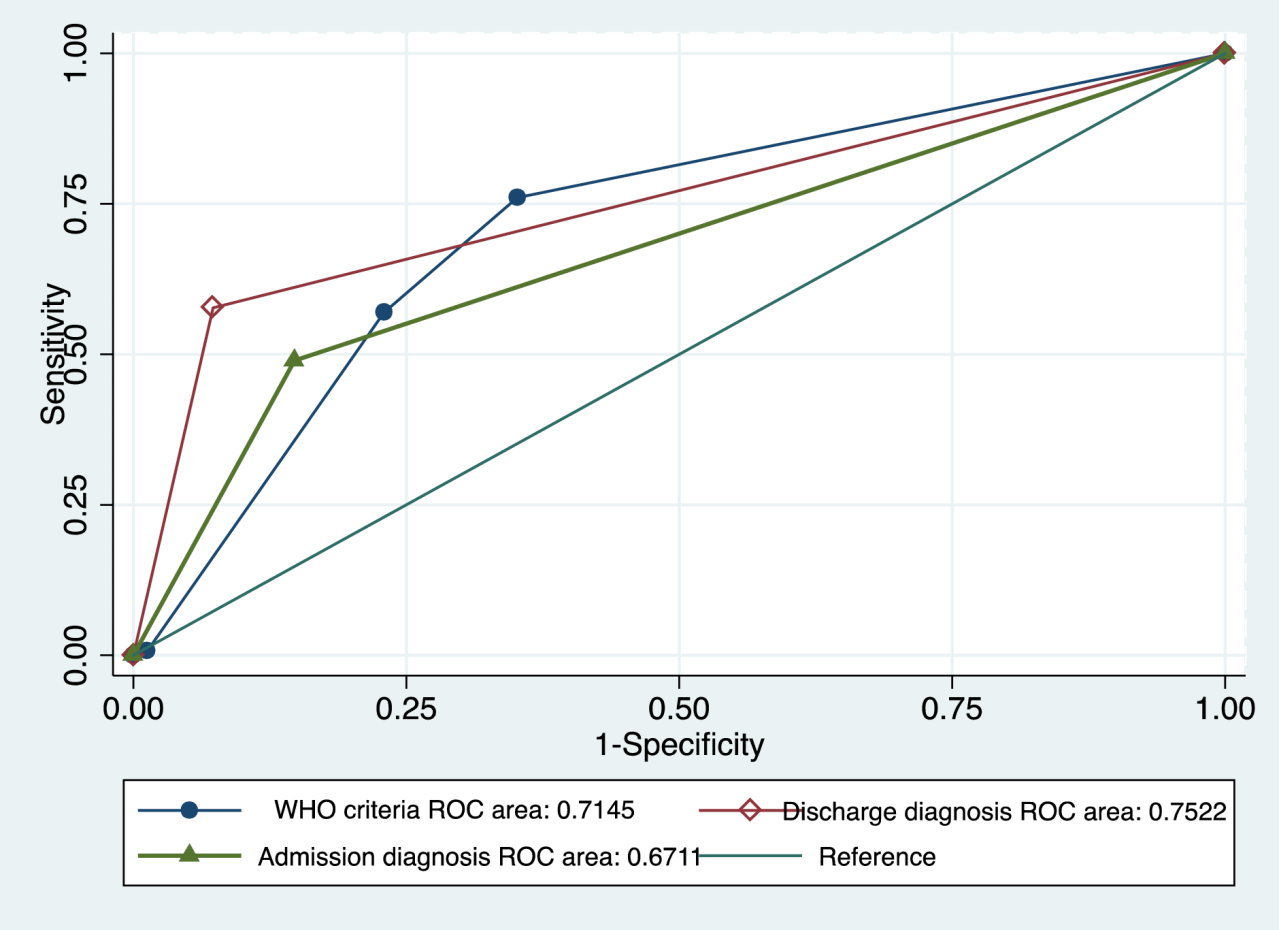
Receiver operating characteristic (ROC) curves of the performance of the WHO classification criteria for dengue and the treating physician’s clinical diagnosis at admission and at discharge for the identification of laboratory-confirmed acute dengue in children and adults enrolled with acute febrile illness in southern Sri Lanka, 2012–2013.

The 2009 World Health Organization (WHO) clinical classification had similar sensitivity for diagnosis of acute dengue in adults vs children (78% vs. 70%, p = 0.09), but had improved sensitivity in secondary vs primary dengue (84% vs. 59%, p < .001). Overall accuracy of the WHO classification in identifying acute dengue was 71.5% (95% CI 68.3–74.6; Fig 1). Of 295 laboratory-confirmed patients with dengue who met the 2009 WHO criteria for dengue, 218 (73.9%) were identified as having dengue with warning signs, 74 (25.1%) without warning signs, and 3 (1.0%) severe dengue. Children with acute dengue identified by WHO criteria were no more likely to be classified as having dengue with warning signs than were adults (84.5% versus 72.6%, respectively, p = 0.06). Similar proportions of patients with primary and secondary dengue were classified as having dengue with warning signs (75.4% versus 74.1%, respectively), although all 3 patients classified as having severe dengue had secondary dengue.

## Discussion

Early confirmation of acute dengue and identification of those at increased risk for severe dengue is desirable to decrease mortality; however, accurate point-of-care diagnostic tools are not widely available across the tropics: therefore, validated clinical instruments to improve case detection and classification are needed. To better identify patients with dengue, including severe dengue, the WHO published updated criteria for the clinical classification of dengue in 2009. However, the sensitivity and specificity of the revised criteria is not known. The presence of epidemic dengue, reproducible enrollment criteria, and rigorous laboratory confirmation supported by excellent follow-up allowed us to retrospectively compare the 2009 WHO criteria for diagnosis of acute dengue versus clinicians’ clinical diagnosis on admission and at discharge. To our knowledge, this is the first evaluation of the performance of the 2009 WHO dengue classification criteria at identifying dengue among hospitalized children and adults with undifferentiated fever in the Southern Province, Sri Lanka.

In our study, patients with acute dengue were more likely to have joint pain, muscle pain, anorexia, right upper quadrant tenderness, rash, leukopenia, thrombocytopenia, and elevated transaminases compared with those with other AFI. Leukopenia and thrombocytopenia were the independent predictors most strongly associated with dengue. Body aches and joint pain were also found to be more common in patients with dengue vs. other AFI in Puerto Rico [8]. A study in Brazil also identified history of rash but additionally taste disorder, conjunctival redness, and lymph node enlargement [9]. Multiple studies have found leukopenia, thrombocytopenia, and elevated transaminases positively associated with confirmed acute dengue [3, 9–14]. Although cough and crackles were less common in patients with acute dengue in the present study, more than one-third of patients with dengue in our cohort reported cough. Similar results were reported in a 1986 epidemic in Puerto Rico [15]. Therefore, respiratory symptoms should not be used to rule out dengue. In our 2007 study, dengue was associated with the absence of sore throat and presence of diarrhea, conjunctivitis, jaundice, abdominal pain, leukopenia, and thrombocytopenia [16]. The small difference in symptoms in the current study compared to 2007 may reflect the infecting DENV serotype, the greater proportion of children and patients with secondary dengue, or other factors. Presence of sore throat was independently associated with acute dengue in this study but only in children, which may explain why presence of sore throat was not associated with acute dengue in our previous study focused on adults.

In this study, enrollment of substantive numbers of children in addition to adults allowed us to perform multivariable analyses to identity features independently associated with acute dengue vs other AFI separately in adults vs children. On multivariable analyses, both adults and children with acute dengue were more likely than those with other AFI to have leukopenia and thrombocytopenia. Additionally, adults were more likely than those with other AFI to have joint pain, higher temperatures, right upper quadrant abdominal pain, and absence of crackles on examination whereas children with dengue were more likely than others to have sore throat, fatigue, oliguria, an elevated hematocrit (≥20% from baseline) and elevated transaminases. Previous reports have suggested that clinical features of dengue may differ in adults vs children; however, most studies have only included adults with dengue [3, 17–20]. Vomiting was associated with dengue vs other AFI in adults in Singapore and abdominal pain with dengue vs other AFI in children in Vietnam [21]; however, other studies have not found abdominal pain to differentiate dengue vs other AFI in adults or in children [22, 23]. Further studies, with sufficient enrollment and objective enrollment (e.g., documented fever) to support controlled analyses, are needed to assess whether the clinical features we found to be independently associated with dengue vs non-dengue AFI in adults and children will be again identified.

We found that several features were much more frequent in secondary dengue than in primary dengue, even in the absence of severe disease. Both adults and children with secondary dengue were more likely to have longer durations of fever prior to enrollment, muscle pain, and thrombocytopenia than patients with primary dengue. Additionally, adults presenting with secondary dengue were more likely to have a lower temperature, vomiting, joint pains, and flushing on examination than adults with primary dengue whereas children with secondary dengue were more likely to have rhinitis/congestion than those with primary dengue. On multivariable analysis, presence of joint pain and thrombocytopenia and absence of cough were independently associated with secondary vs primary dengue in adults and no features were identified in children. Studies comparing secondary vs primary dengue in either adults or children are lacking [8, 23–25]. In a cross-sectional study in Vietnam, Phuong *et al* found no clinical differences in a cohort of 202 patients with secondary and 32 with primary dengue [23]. However, Gregory et al found that those with secondary dengue were more likely to have body aches, joint pain, nausea, and vomiting compared with patients with primary dengue [8]. In Thailand, Pancharoen et al found that children with primary dengue were more likely to have runny nose, diarrhea, rash, and seizure and less commonly headache, vomiting, and abdominal pain [24]. Of note, we found that most (70%) patients had secondary dengue, as would be expected given progressively larger island-wide, annual dengue epidemics; however, disease severity was generally low [26]. This low severity may be related to the enrollment of a cohort with undifferentiated fever, rather than biased enrollment of patients with classic signs of severe dengue/dengue hemorrhagic fever/ dengue shock syndrome. DENV-1 is generally considered to cause less severe disease than DENV-2 and -3 but disease severity is also influenced by the strain of virus within a serotype. Large population-based studies are needed to determine if features of secondary dengue also independently predict severe disease and to delineate the influence of virus serotype and strain on disease severity.

We found that the sensitivity of the treating physician’s clinical diagnosis of dengue at the time of admission was improved relative to our 2007 study (58 vs. 14%), which likely reflects heightened clinical suspicion owing to our prior study, the 2009 classification criteria, and/or the epidemic transmission in 2012. However, clinical diagnosis on admission was less sensitive than the 2009 WHO classification. In contrast, the treating physician’s clinical diagnosis of dengue at the time of discharge was more sensitive than the WHO classification, as would be expected given the opportunity for clinical observation and non-etiologic laboratory investigations. The WHO classification also had higher sensitivity for diagnosis of secondary vs. primary dengue (84% vs. 59%, p < .001, respectively). Wanigasuriya et al found that the 2009 classification better identified dengue with warning signs compared with the 1997 WHO classification and Jayaratne et al found that ≥ 5 warning signs predicted severe dengue [27, 28]. Others have suggested that the 2009 criteria may overestimate disease severity [6, 29–33]. Although the 2009 WHO classification had relatively high sensitivity in our cohort, the criteria classified almost 75% as having warning signs, even though <10% developed plasma leakage or bleeding. Notably the 25% without warning signs per classification might have not required hospital admission. Since dengue with warning signs is thought to require monitoring in hospital and many patients without warning signs hospitalized, possible overestimation of dengue disease severity has important implications for an already overburdened public healthcare system.

Sri Lanka has experienced annual, island-wide large epidemics of dengue in the past decade with increasing numbers of patients being hospitalized [26]. Our study describes the clinical features and performance of the WHO classification criteria among children and adults admitted with undifferentiated fever to the largest tertiary care hospital in the Southern Province. Sri Lanka has a longstanding and effective public health system for the control of communicable diseases, with achievements that include the elimination of malaria, high coverage of the population with childhood vaccinations, and very low prevalence of human immunodeficiency virus [34]. No comprehensive studies detail the etiologies of acute febrile illness, but the annual health bulletin lists notifiable diseases such as dengue, varicella, mumps, dysentery, leptospirosis, viral hepatitides, and enteric fever as being common [34]. Our prior studies in the Southern Province have shown the relatively high prevalence of leptospirosis and rickettsial illnesses as causes of fever among hospitalized patients in the same hospital [35, 36].

In the current study, we only enrolled patients hospitalized for AFI, so we could not evaluate features associated with hospitalization; however, 92.6% of those with acute dengue were hospitalized in our prior study [16]. The relatively small proportion of patients with objective indices of severe disease may be explained by the fact that we enrolled at a public tertiary care hospital where patients may have been referred and admitted for reasons other than disease severity. The frequency of hospitalization with acute dengue is consistent with Sri Lankan guidelines to hospitalize those with dengue and platelet counts ≤ 100,000, and with WHO 2009 guidelines recommending hospitalization for those with dengue with warning signs. Comparisons of clinical features of dengue in children are complicated by underestimation of subjective symptoms in small children (ascertainment bias); however, the median age of children with acute dengue in this study was 11 years and we identified several features as suggestive of dengue. Our estimates of secondary dengue are conservative, since we required virologic evidence to distinguish secondary acute dengue from past dengue and 10% of patients with acute dengue had insufficient acute phase sera for IgG ELISA. Additionally, we did not perform or record tourniquet tests, since earlier studies have suggested that the utility of the tourniquet test is reduced in patients with darker skin and many studies have excluded this test in evaluations of the performance of the 2009 WHO dengue diagnostic criteria [37]. We may, therefore, have slightly underestimated the sensitivity of the 2009 WHO criteria, which includes a positive tourniquet test as suggestive of probable dengue [5, 38]. We may also have underestimated mortality if patients died before being able to come to hospital despite free medical care. Finally, our study was conducted in a specific population of children and adults admitted with undifferentiated fever to the largest tertiary care hospital in one province of Sri Lanka. We used reproducible enrollment criteria and rigorous laboratory confirmation, but our findings may not be generalizable to other regions in Sri Lanka or to outpatient populations. Although a tertiary care center, this hospital provides both primary and tertiary care to a large portion of the population in the Southern Province (only approximately 6% of hospitals admissions during 2012–2013 were due to transfers, per hospital data), thus provides a good representation of the general population in the area.

In conclusion, we identified clinical features independently associated with dengue vs other AFI in adults vs children in a large cohort of patients enrolled with acute febrile illness. We found high sensitivity but low specificity of the 2009 WHO dengue classification system vs. clinical diagnosis at presentation. Strengths of our study include reproducible, unbiased, prospectively applied enrollment criteria, large sample size (for children in addition to adults), and rigorous laboratory confirmation of dengue supported by 90% convalescent follow-up. This rigorous study design allowed us to evaluate the performance of the most recent (2009) WHO clinical criteria vs clinical diagnosis in those with reference standard-confirmed acute dengue, including subsets of adults vs children and those with secondary vs primary dengue. Most dengue cases during this epidemic were relatively mild with few cases of severe dengue and no deaths, despite a predominance of secondary dengue and dengue with warning signs according to the 2009 WHO classification. It is possible that different results could be seen in other geographic regions wherein particularly the non- dengue AFI group may differ. Additional prospective studies of dengue, enrolling patients with AFI using standardized protocols with reproducible enrollment criteria, will be needed to fully assess the predictive capacity of specific clinical features in adults vs children and secondary vs primary dengue, in addition to clinical outcomes and costs associated with use of the 2009 WHO classification for management of patients with acute dengue worldwide.

## Supporting information

S1 Checklist STROBE checklist. (DOC)

S1 Fig (TIF)
