# Supplementary figures and images for: Evaluation of the WHO 2009 classification for diagnosis of acute dengue in a large cohort of adults and children in Sri Lanka during a dengue-1 epidemic
Source: PLoS Negl Trop Dis. 2018 Feb 9;12(2):e0006258. doi: 10.1371/journal.pntd.0006258 (PMC5823472; doi:10.1371/journal.pntd.0006258)

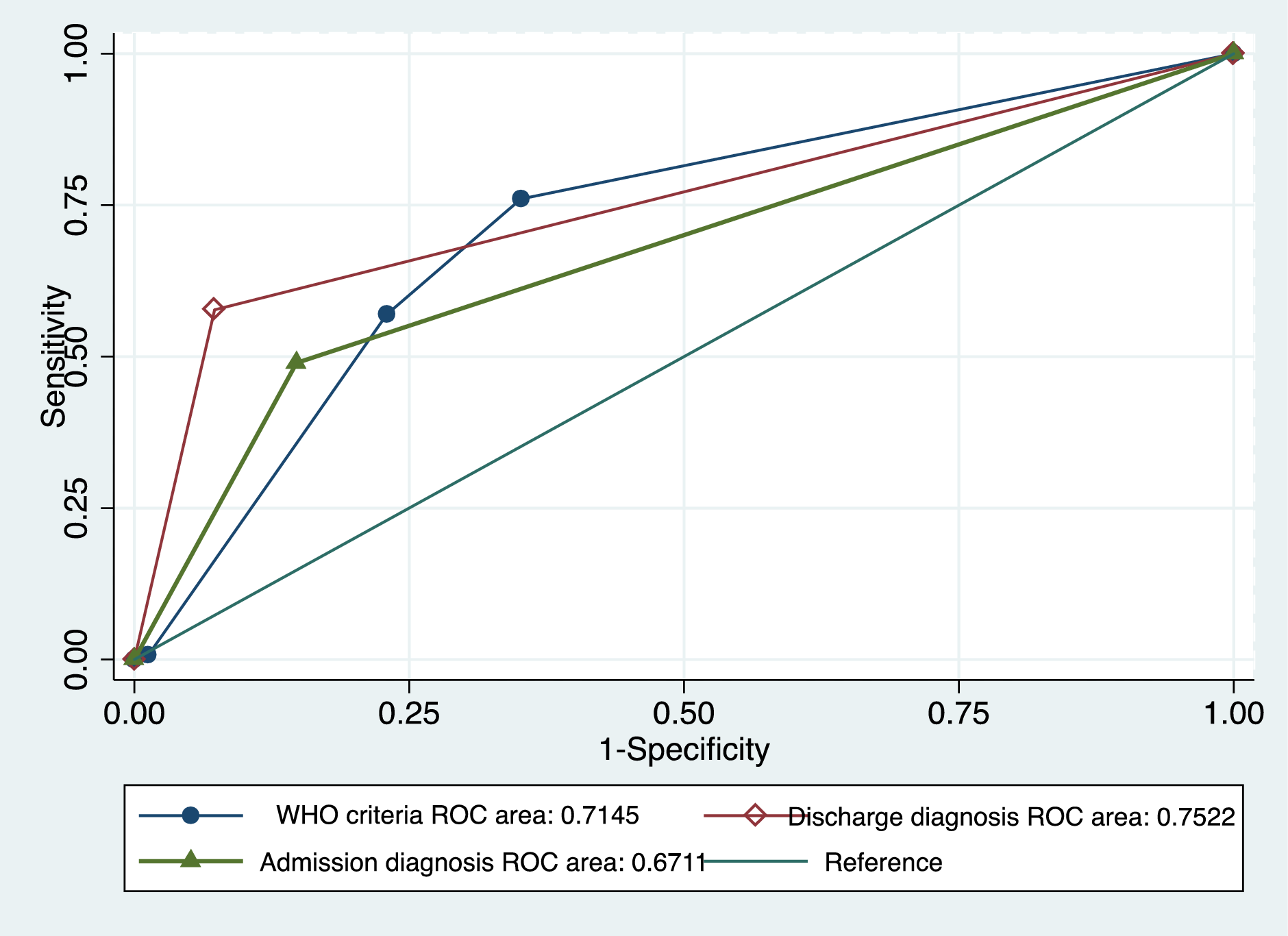

Supplement: S1 Fig — (TIF) [file pntd.0006258.s002.tif]
